# Bis(4-dimethyl­amino-1-ethyl­pyridinium) bis­(1,2-dicyano­ethene-1,2-dithiol­ato-κ^2^ *S*,*S*′)nickelate(II)

**DOI:** 10.1107/S1600536812008161

**Published:** 2012-03-10

**Authors:** Shan-Shan Yu, Hong Zhou, Xiao-Ming Ren

**Affiliations:** a College of Science, Nanjing University of Technology, Nanjing 210009, People’s Republic of China; b School of Biochemical and Environmental Engineering, Nanjing Xiaozhuang College, Nanjing 210017, People’s Republic of China

## Abstract

The asymmetric unit of the title complex, (C_9_H_15_N_2_)_2_[Ni(C_4_N_2_S_2_)_2_], comprises one 4-dimethyl­amino-1-ethyl­pyri­din­ium cation and one half of a [Ni(mnt)_2_]^2−^ (mnt^2−^ = maleo­nitrile­dithiol­ate) anion; the complete anion is generated by the application of a centre of inversion. The Ni^II^ ion is coordinated by four S atoms of two mnt^2−^ ligands and exhibits a square-planar coordination geometry.

## Related literature

For the magnetic and conducting properties of related complexes, see: Belo & Almedia (2010 ▶); Nishijo *et al.* (2000 ▶); Duan *et al.* (2010 ▶); Ni *et al.* (2005 ▶). For novel magnetic behaviour, see: Ni *et al.* (2004 ▶); Ren *et al.* (2004 ▶). For a related [Ni(mnt)_2_]^2−^ complex, see: Yao *et al.* (2008 ▶). For the synthesis of the starting materials, see: Davison & Holm (1967 ▶); Duan *et al.* (2011 ▶).
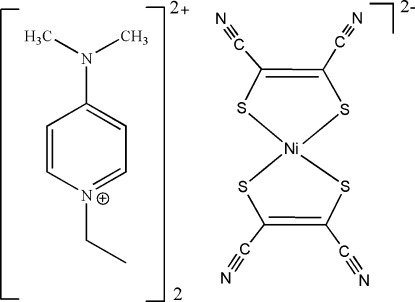


## Experimental

### 

#### Crystal data

(C_9_H_15_N_2_)_2_[Ni(C_4_N_2_S_2_)_2_]
*M* *_r_* = 641.55
Triclinic, 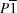

*a* = 8.1468 (14) Å
*b* = 9.3305 (16) Å
*c* = 11.663 (3) Å
α = 108.243 (3)°
β = 100.034 (3)°
γ = 107.830 (2)°
*V* = 765.0 (3) Å^3^
*Z* = 1
Mo *K*α radiation
μ = 0.94 mm^−1^
*T* = 296 K
0.3 × 0.1 × 0.1 mm

#### Data collection

Bruker SMART CCD area-detector diffractometer
Absorption correction: multi-scan (*SADABS*; Sheldrick, 2002 ▶) *T*_min_ = 0.894, *T*_max_ = 0.910
5798 measured reflections
2827 independent reflections
2371 reflections with *I* > 2σ(*I*)
*R*_int_ = 0.031

#### Refinement

*R*[*F* ^2^ > 2σ(*F* ^2^)] = 0.037
*wR*(*F* ^2^) = 0.128
*S* = 0.95
2827 reflections
181 parameters
H-atom parameters constrained
Δρ_max_ = 0.25 e Å^−3^
Δρ_min_ = −0.34 e Å^−3^

### 

Data collection: *SMART* (Bruker, 2000 ▶); cell refinement: *SAINT* (Bruker, 2000 ▶); data reduction: *SAINT*; program(s) used to solve structure: *SHELXTL* (Sheldrick, 2008 ▶); program(s) used to refine structure: *SHELXTL*; molecular graphics: *SHELXTL*; software used to prepare material for publication: *SHELXTL*.

## Supplementary Material

Crystal structure: contains datablock(s) global, I. DOI: 10.1107/S1600536812008161/tk5062sup1.cif

Structure factors: contains datablock(s) I. DOI: 10.1107/S1600536812008161/tk5062Isup2.hkl

Additional supplementary materials:  crystallographic information; 3D view; checkCIF report

## Figures and Tables

**Table:** 

Ni1—S2	2.1776 (8)
Ni1—S1	2.1794 (8)

**Table:** 

S2—Ni1—S1	88.00 (3)

**Fig. 1.**
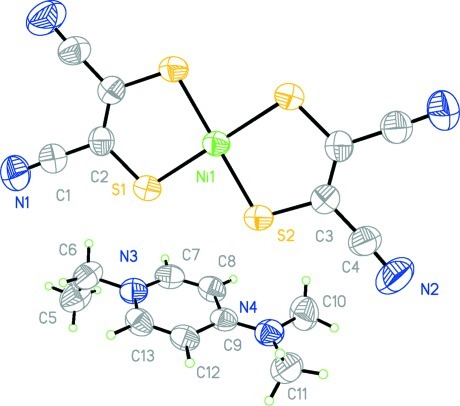
The molecular structure of (I), showing the atom-numbering scheme and displacement ellipsoids at the 30% probability level. Unlabelled atoms are related by the symmetry operation 2-x, 1-y, 1-z.

## supplementary crystallographic information

### Comment

Bis-1,2-dithiolene complexes of transition metals have been widely studied due to their novel properties in the areas of magnetic and conducting materials for example (Belo & Almedia, 2010; Nishijo *et al.*, 2000; Duan *et al.*, 2010; Ni *et al.*, 2005). The mesomorphous neutral nickel-dithiolene complexes, with a focus on aspects of crystalline to liquid crystal transition behaviour has attracted attention and our research focus has been to try to design and assemble ionic and planar nickel-dithiolene mesogens with novel magnetic behaviour (Ni *et al.*, 2004; Ren *et al.*, 2004). Herein, we report the crystal structure of the title complex (I).

The molecular structure of (I) is illustrated in Fig. 1. and selected bond lengths and bond angles are given in Table 1. Complex (I) crystallizes in the triclinic space group *P*1 at 293 K and the asymmetric units comprises one half of a [Ni(mnt)_2_]^2-^ anion and one 1-ethyl-4-*N*,*N*-dimethylpyridinium cation. The Ni^II^ ion in the centrosymmetric [Ni(mnt)_2_]^2-^ anion is coordinated by four sulfur atoms of two mnt^2-^ ligands, and exhibits square-planar coordination geometry. Bond lengths and angles of the anion are in good agreement with the other [Ni(mnt)_2_]^2-^ compounds (*e.g*. Yao *et al.*, 2008). In the crystal packing, the cations and anions are arranged in alternate layers, which are parallel to *bc* plane.

### Experimental

All reagents and chemicals were purchased from commercial sources and used without further purification. The staring materials disodium maleonitriledithiolate, and 1-ethyl-4-*N*,*N*-dimethylpyridinium bromide were synthesized following the literature procedures (Davison & Holm, 1967; Duan *et al.*, 2011). Disodium maleonitriledithiolate (456 mg, 2.5 mmol) and nickel chloride hexahydrate (297 mg, 1.25 mmol) were mixed under stirring in water (20 ml) at room temperature. Subsequently, a solution of 1-ethyl-4-*N*,*N*-dimethylpyridinium bromide (2.5 mmol) in water (10 ml) was added to the mixture, and the red precipitate that was immediately formed was filtered off and washed with water. The crude product was recrystallized in acetone to give red blocks.

### Refinement

Carbon-bound H-atoms were placed in calculated positions [C—H 0.93 to 0.97 Å, *U*_iso_(H) 1.2 to 1.5*U*_eq_(C)] and were included in the refinement in the riding model approximation.

### Crystal data

**Table:**

(C_9_H_15_N_2_)_2_[Ni(C_4_N_2_S_2_)_2_]	*V* = 765.0 (3) Å^3^
*M**_r_* = 641.55	*Z* = 1
Triclinic, *P*1	*F*(000) = 334
Hall symbol: -P 1	*D*_x_ = 1.393 Mg m^−^^3^
*a* = 8.1468 (14) Å	Mo *K*α radiation, λ = 0.71073 Å
*b* = 9.3305 (16) Å	µ = 0.94 mm^−^^1^
*c* = 11.663 (3) Å	*T* = 296 K
α = 108.243 (3)°	Block, red
β = 100.034 (3)°	0.3 × 0.1 × 0.1 mm
γ = 107.830 (2)°	

### Data collection

**Table:**

Bruker SMART CCD area-detector diffractometer	2827 independent reflections
Radiation source: fine-focus sealed tube	2371 reflections with *I* > 2σ(*I*)
Graphite monochromator	*R*_int_ = 0.031
φ and ω scans	θ_max_ = 25.5°, θ_min_ = 1.9°
Absorption correction: multi-scan (*SADABS*; Sheldrick, 2002)	*h* = −9→9
*T*_min_ = 0.894, *T*_max_ = 0.910	*k* = −11→11
5798 measured reflections	*l* = −14→14

### Refinement

**Table:**

Refinement on *F*^2^	Primary atom site location: structure-invariant direct methods
Least-squares matrix: full	Secondary atom site location: difference Fourier map
*R*[*F*^2^ > 2σ(*F*^2^)] = 0.037	Hydrogen site location: inferred from neighbouring sites
*wR*(*F*^2^) = 0.128	H-atom parameters constrained
*S* = 0.95	*w* = 1/[σ^2^(*F*_o_^2^) + (0.091*P*)^2^ + 0.1169*P*] where *P* = (*F*_o_^2^ + 2*F*_c_^2^)/3
2827 reflections	(Δ/σ)_max_ < 0.001
181 parameters	Δρ_max_ = 0.25 e Å^−^^3^
0 restraints	Δρ_min_ = −0.34 e Å^−^^3^

### Special details

**Table:**

Geometry. All e.s.d.'s (except the e.s.d. in the dihedral angle between two l.s. planes) are estimated using the full covariance matrix. The cell e.s.d.'s are taken into account individually in the estimation of e.s.d.'s in distances, angles and torsion angles; correlations between e.s.d.'s in cell parameters are only used when they are defined by crystal symmetry. An approximate (isotropic) treatment of cell e.s.d.'s is used for estimating e.s.d.'s involving l.s. planes.
Refinement. Refinement of *F*^2^ against ALL reflections. The weighted *R*-factor *wR* and goodness of fit *S* are based on *F*^2^, conventional *R*-factors *R* are based on *F*, with *F* set to zero for negative *F*^2^. The threshold expression of *F*^2^ > σ(*F*^2^) is used only for calculating *R*-factors(gt) *etc*. and is not relevant to the choice of reflections for refinement. *R*-factors based on *F*^2^ are statistically about twice as large as those based on *F*, and *R*- factors based on ALL data will be even larger.

### Fractional atomic coordinates and isotropic or equivalent isotropic displacement parameters (Å^2^)

**Table:**

	*x*	*y*	*z*	*U*_iso_*/*U*_eq_	
Ni1	1.0000	0.5000	0.5000	0.04490 (19)	
S1	0.82193 (11)	0.48140 (9)	0.32858 (7)	0.0569 (2)	
S2	0.98454 (10)	0.73406 (8)	0.59749 (7)	0.0544 (2)	
N1	0.6004 (5)	0.2159 (4)	−0.0092 (3)	0.0933 (10)	
N2	1.1487 (5)	1.0752 (4)	0.9081 (3)	0.1059 (12)	
N3	0.4538 (3)	0.6143 (3)	0.2736 (2)	0.0612 (6)	
C9	0.6202 (4)	0.8572 (3)	0.5178 (3)	0.0537 (6)	
C1	0.6864 (5)	0.2485 (4)	0.0912 (3)	0.0653 (8)	
C2	0.7958 (4)	0.2948 (3)	0.2175 (3)	0.0524 (6)	
C3	1.1208 (4)	0.7989 (3)	0.7514 (3)	0.0514 (6)	
C4	1.1395 (4)	0.9529 (4)	0.8407 (3)	0.0664 (8)	
C5	0.1777 (5)	0.4619 (5)	0.0912 (4)	0.0947 (12)	
H5A	0.1089	0.4361	0.1461	0.142*	
H5B	0.1256	0.3745	0.0089	0.142*	
H5C	0.1762	0.5614	0.0844	0.142*	
C6	0.3668 (5)	0.4828 (4)	0.1439 (3)	0.0798 (10)	
H6A	0.4360	0.5097	0.0884	0.096*	
H6B	0.3682	0.3805	0.1468	0.096*	
C7	0.4085 (4)	0.5897 (4)	0.3731 (3)	0.0648 (8)	
H7	0.3215	0.4893	0.3593	0.078*	
C8	0.4840 (4)	0.7048 (4)	0.4930 (3)	0.0634 (8)	
H8	0.4462	0.6834	0.5589	0.076*	
N4	0.7030 (4)	0.9716 (3)	0.6353 (2)	0.0637 (6)	
C10	0.6603 (6)	0.9431 (5)	0.7439 (3)	0.0926 (11)	
H10A	0.5331	0.9145	0.7325	0.139*	
H10B	0.7268	1.0406	0.8190	0.139*	
H10C	0.6925	0.8554	0.7523	0.139*	
C11	0.8478 (5)	1.1248 (4)	0.6603 (3)	0.0776 (9)	
H11A	0.9461	1.1030	0.6341	0.116*	
H11B	0.8891	1.1914	0.7493	0.116*	
H11C	0.8042	1.1815	0.6142	0.116*	
C12	0.6630 (4)	0.8805 (3)	0.4110 (3)	0.0567 (7)	
H12	0.7490	0.9796	0.4213	0.068*	
C13	0.5803 (4)	0.7602 (4)	0.2938 (3)	0.0599 (7)	
H13	0.6116	0.7786	0.2252	0.072*	

### Atomic displacement parameters (Å^2^)

**Table:**

	*U*^11^	*U*^22^	*U*^33^	*U*^12^	*U*^13^	*U*^23^
Ni1	0.0457 (3)	0.0453 (3)	0.0501 (3)	0.0200 (2)	0.0176 (2)	0.0227 (2)
S1	0.0658 (5)	0.0571 (4)	0.0549 (4)	0.0324 (4)	0.0152 (3)	0.0238 (3)
S2	0.0605 (4)	0.0485 (4)	0.0584 (4)	0.0259 (3)	0.0153 (3)	0.0228 (3)
N1	0.105 (2)	0.106 (2)	0.0599 (18)	0.047 (2)	0.0083 (17)	0.0229 (17)
N2	0.119 (3)	0.070 (2)	0.105 (3)	0.0448 (19)	0.021 (2)	0.0028 (18)
N3	0.0563 (14)	0.0638 (15)	0.0696 (16)	0.0287 (12)	0.0214 (12)	0.0273 (13)
C9	0.0549 (16)	0.0583 (15)	0.0637 (17)	0.0323 (13)	0.0240 (13)	0.0302 (14)
C1	0.072 (2)	0.0681 (18)	0.0600 (19)	0.0303 (16)	0.0212 (16)	0.0262 (15)
C2	0.0498 (15)	0.0568 (15)	0.0490 (15)	0.0174 (12)	0.0174 (12)	0.0205 (12)
C3	0.0507 (15)	0.0496 (14)	0.0544 (16)	0.0160 (12)	0.0209 (12)	0.0215 (12)
C4	0.0656 (19)	0.0556 (17)	0.075 (2)	0.0259 (14)	0.0180 (16)	0.0202 (16)
C5	0.072 (2)	0.079 (2)	0.096 (3)	0.0212 (19)	0.001 (2)	0.008 (2)
C6	0.079 (2)	0.069 (2)	0.081 (2)	0.0338 (18)	0.0181 (18)	0.0127 (17)
C7	0.0547 (17)	0.0584 (17)	0.086 (2)	0.0189 (14)	0.0233 (16)	0.0355 (16)
C8	0.0620 (18)	0.0736 (19)	0.073 (2)	0.0286 (15)	0.0327 (16)	0.0427 (17)
N4	0.0721 (16)	0.0657 (15)	0.0679 (16)	0.0372 (13)	0.0305 (13)	0.0289 (13)
C10	0.113 (3)	0.110 (3)	0.065 (2)	0.051 (2)	0.041 (2)	0.032 (2)
C11	0.082 (2)	0.0630 (19)	0.078 (2)	0.0297 (17)	0.0146 (18)	0.0192 (17)
C12	0.0554 (16)	0.0566 (15)	0.0668 (18)	0.0200 (13)	0.0229 (14)	0.0341 (14)
C13	0.0572 (17)	0.0734 (18)	0.0645 (18)	0.0293 (15)	0.0264 (14)	0.0380 (16)

### Geometric parameters (Å, º)

**Table:**

Ni1—S2	2.1776 (8)	C5—H5A	0.9600
Ni1—S2^i^	2.1776 (8)	C5—H5B	0.9600
Ni1—S1^i^	2.1794 (8)	C5—H5C	0.9600
Ni1—S1	2.1794 (8)	C6—H6A	0.9700
S1—C2	1.738 (3)	C6—H6B	0.9700
S2—C3	1.742 (3)	C7—C8	1.358 (4)
N1—C1	1.147 (4)	C7—H7	0.9300
N2—C4	1.136 (4)	C8—H8	0.9300
N3—C7	1.342 (4)	N4—C11	1.452 (4)
N3—C13	1.351 (4)	N4—C10	1.451 (4)
N3—C6	1.493 (4)	C10—H10A	0.9600
C9—N4	1.339 (4)	C10—H10B	0.9600
C9—C8	1.415 (4)	C10—H10C	0.9600
C9—C12	1.412 (4)	C11—H11A	0.9600
C1—C2	1.435 (4)	C11—H11B	0.9600
C2—C3^i^	1.354 (4)	C11—H11C	0.9600
C3—C2^i^	1.354 (4)	C12—C13	1.356 (4)
C3—C4	1.431 (4)	C12—H12	0.9300
C5—C6	1.483 (5)	C13—H13	0.9300
			
S2—Ni1—S2^i^	180.0	C5—C6—H6B	109.2
S2—Ni1—S1^i^	92.00 (3)	N3—C6—H6B	109.2
S2^i^—Ni1—S1^i^	88.00 (3)	H6A—C6—H6B	107.9
S2—Ni1—S1	88.00 (3)	N3—C7—C8	122.9 (3)
S2^i^—Ni1—S1	92.00 (3)	N3—C7—H7	118.5
S1^i^—Ni1—S1	180.000 (1)	C8—C7—H7	118.5
C2—S1—Ni1	103.04 (10)	C7—C8—C9	120.0 (3)
C3—S2—Ni1	103.41 (10)	C7—C8—H8	120.0
C7—N3—C13	118.5 (3)	C9—C8—H8	120.0
C7—N3—C6	120.5 (3)	C9—N4—C11	121.5 (3)
C13—N3—C6	121.0 (3)	C9—N4—C10	121.3 (3)
N4—C9—C8	121.9 (3)	C11—N4—C10	117.0 (3)
N4—C9—C12	122.3 (3)	N4—C10—H10A	109.5
C8—C9—C12	115.8 (3)	N4—C10—H10B	109.5
N1—C1—C2	178.1 (3)	H10A—C10—H10B	109.5
C3^i^—C2—C1	122.2 (3)	N4—C10—H10C	109.5
C3^i^—C2—S1	121.3 (2)	H10A—C10—H10C	109.5
C1—C2—S1	116.5 (2)	H10B—C10—H10C	109.5
C2^i^—C3—C4	122.6 (3)	N4—C11—H11A	109.5
C2^i^—C3—S2	120.3 (2)	N4—C11—H11B	109.5
C4—C3—S2	117.2 (2)	H11A—C11—H11B	109.5
N2—C4—C3	177.2 (4)	N4—C11—H11C	109.5
C6—C5—H5A	109.5	H11A—C11—H11C	109.5
C6—C5—H5B	109.5	H11B—C11—H11C	109.5
H5A—C5—H5B	109.5	C13—C12—C9	120.8 (3)
C6—C5—H5C	109.5	C13—C12—H12	119.6
H5A—C5—H5C	109.5	C9—C12—H12	119.6
H5B—C5—H5C	109.5	N3—C13—C12	122.0 (3)
C5—C6—N3	112.0 (3)	N3—C13—H13	119.0
C5—C6—H6A	109.2	C12—C13—H13	119.0
N3—C6—H6A	109.2		

Symmetry code: (i) −*x*+2, −*y*+1, −*z*+1.
